# Supplementary material for: Bending and breaking of stripes in a charge ordered manganite
Source: Nat Commun. 2017 Dec 1;8:1883. doi: 10.1038/s41467-017-02156-1 (PMC5709367; doi:10.1038/s41467-017-02156-1)
Supplement: Supplementary file 1 — Supplementary Information [file 41467_2017_2156_MOESM1_ESM.pdf]

# Supplementary Information

## Supplementary Figures

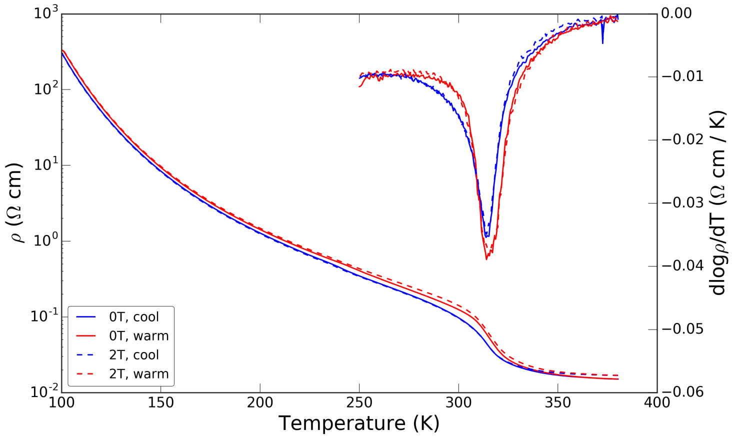


**Supplementary Figure 1: Temperature dependence of the electrical resistivity at 0 T and 2 T.** Electrical resistivity of $Bi_{0.35}Sr_{0.18}Ca_{0.47}MnO_3$ (BSCMO) as a function of temperature and thermal cycling. As shown in the inset, a resistivity anomaly associated with charge-ordering occurs just above room temperature ($315K$) and has thermal cycling dependence (color indicates heating or cooling). Instead of a sharp phase transition, we observe a broad and gradual transition. The charge-ordering critical temperature is seen more clearly in the $\frac{d(\log \rho)}{dt}$ plot. Measurements were repeated in the absence and presence of a $\sim$2 T applied magnetic field comparable to that of the objective lens at the position of the specimen. The critical temperature and functional form of the resistivity anomaly associated with charge-ordering are unchanged at 0 T and $\sim$2 T, suggesting that the charge-ordered phase is intrinsic to the crystal and robust to the applied field. Note that resistivity measurements are performed on single crystals ($\sim$0.5 $\times$ 0.5 mm) with multiple orthorhombic twin domains (typical size $\sim$100$\mu$m). The nominal critical temperature was determined by finding the minima of the four $\frac{d(\log \rho)}{dt}$ curves following a mild Gaussian smoothing, yielding $T_c = 315 \pm 0.5K$.

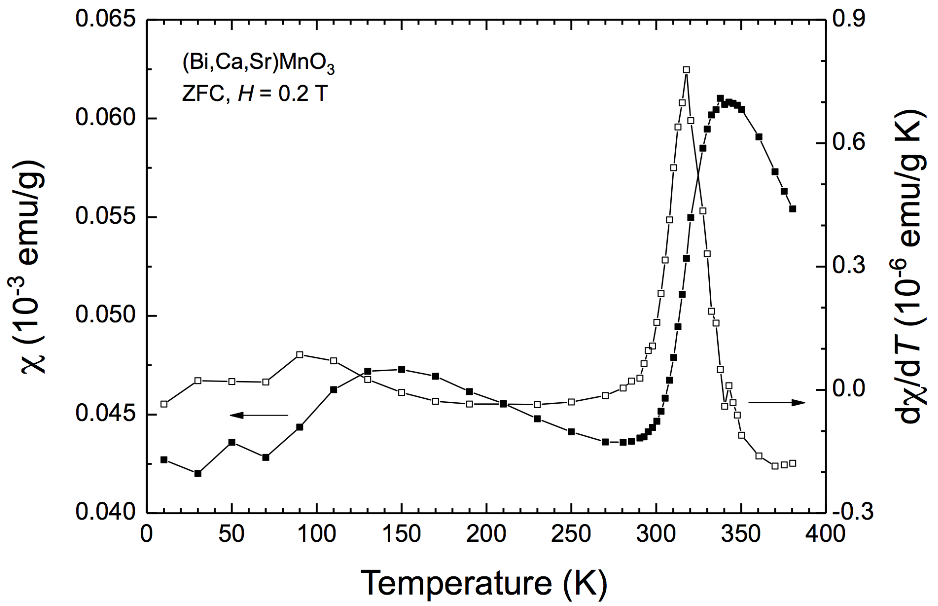


**Supplementary Figure 2: Temperature dependence of the magnetic susceptibility.** Magnetic susceptibility (left axis, filled symbols) and its temperature derivative (right axis, open symbols) of $Bi_{0.35}Sr_{0.18}Ca_{0.47}MnO_3$ as a function of temperature. A phase transition is apparent as a peak in the derivative curve at $T_c \sim 318$ K, which is comparable to that indicated by resistivity measurements.

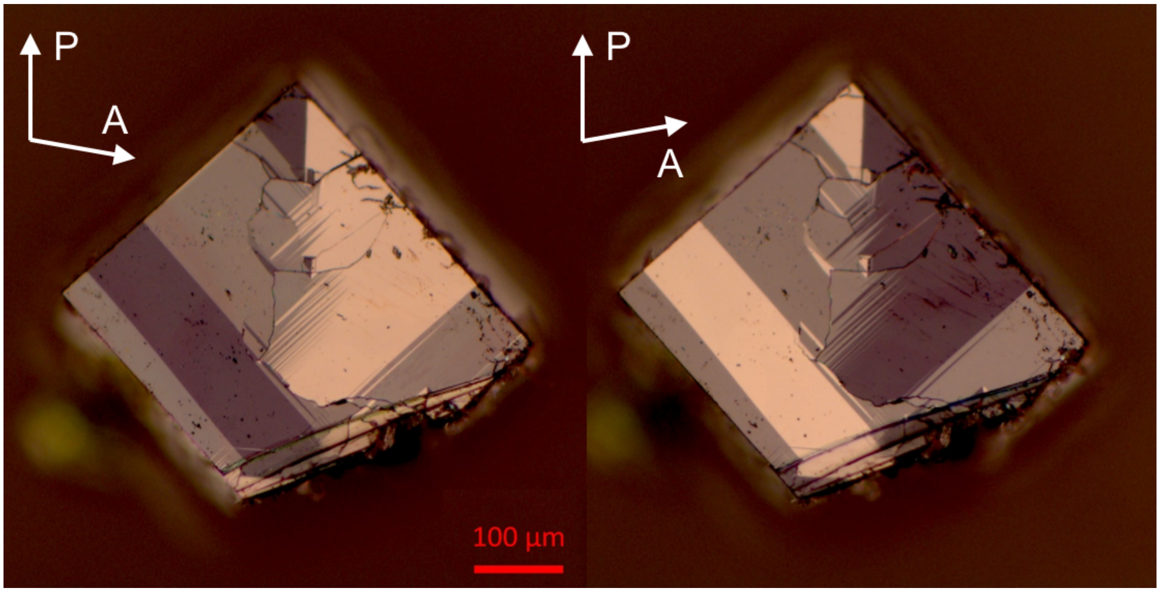


**Supplementary Figure 3: Linear polarized optical microscopy of BSCMO twin domains.** Linear polarized optical microscopy shows orthorhombic twin domains of $\sim$100 $\mu$m extent in the crystals under study. Left and right images are taken under different analyzer angles (A) with fixed light polarization direction (P). In contrast, the electron diffraction data shown corresponds to a $\sim$1 $\mu$m diameter selected area, and the HAADF-STEM data has a $\sim$30 nm field of view. No evidence of twinning exists in any diffraction patterns of the regions examined, indicating the area of study did not include twins of the a/b or b/c axes. Moreover, the STEM data presented here is representative of many datasets acquired over an area of $\sim$2 $\mu$m extent, suggesting that the phenomena we observe cannot be the result of imaging along a twin boundary.

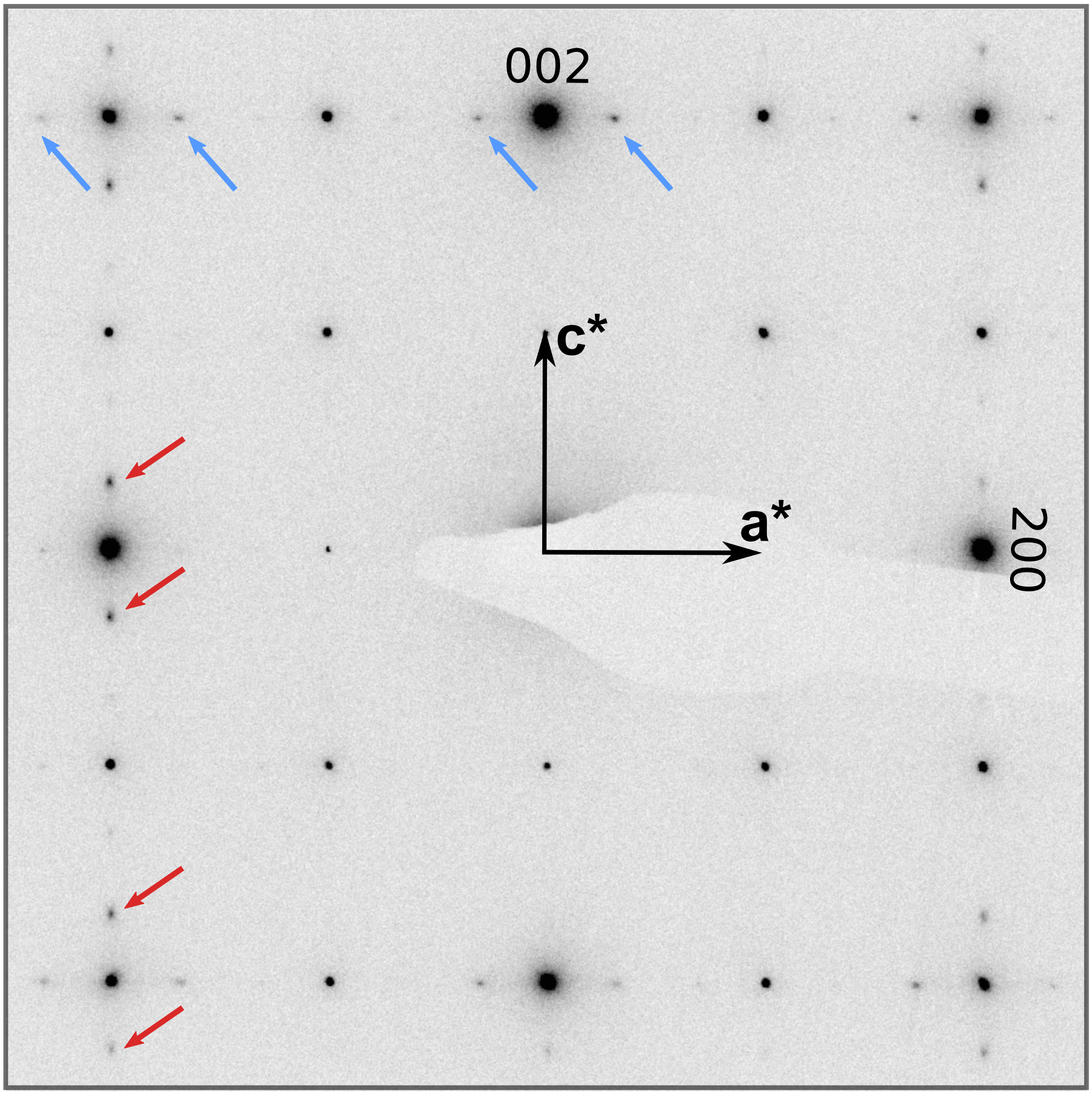


**Supplementary Figure 4: Electron diffraction of BSCMO.** Electron diffraction pattern over $1 \mu m$ area indexed in the $Pnma$ space group. Satellite peaks corresponding to modulations along orthogonal directions are marked by blue and red arrows. Transverse, displacive lattice modulations are indicated.

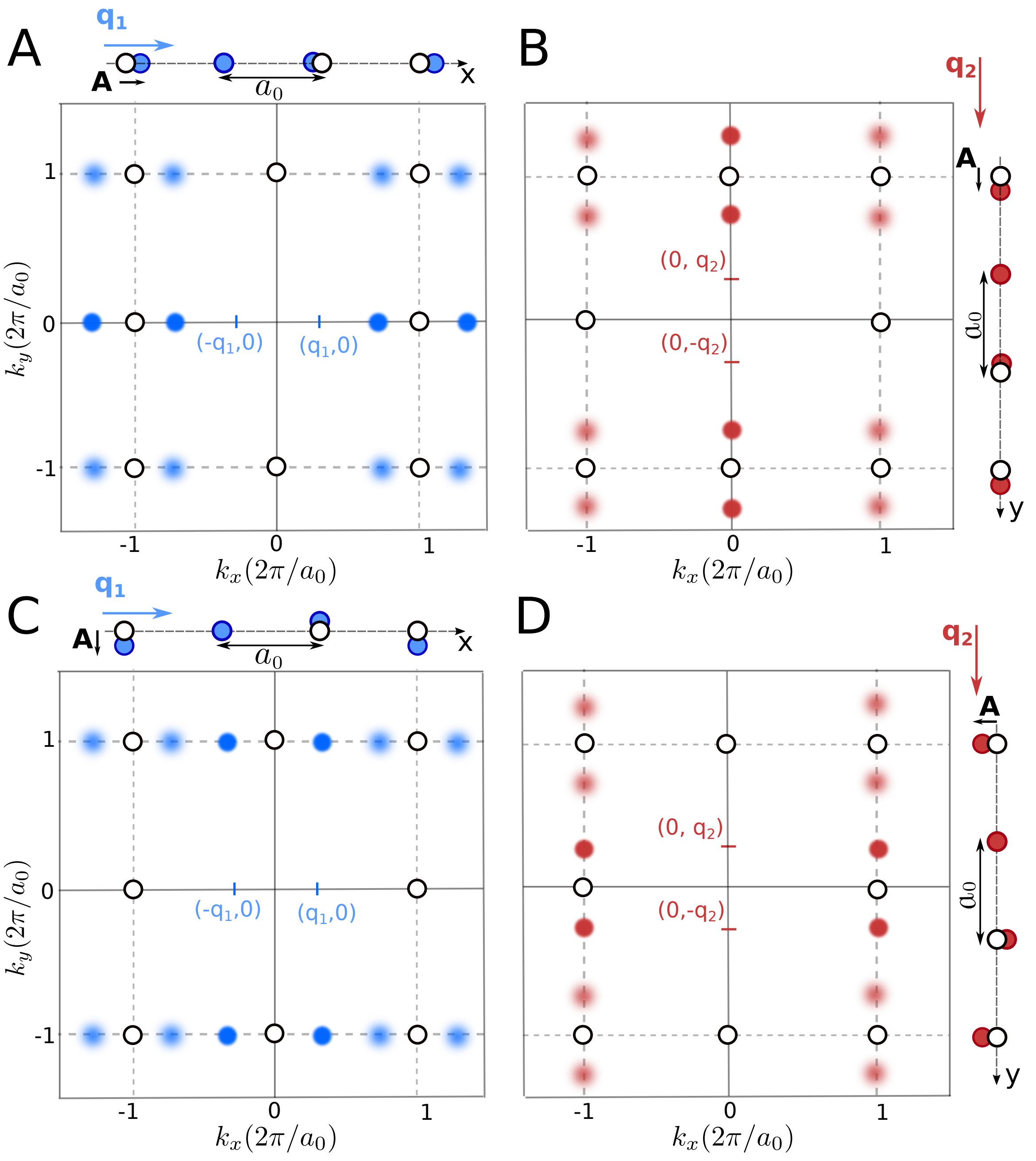


**Supplementary Figure 5: Longitudinal vs. transverse periodic lattice displacements (PLDs) in Fourier space.** (**A,B**) Reciprocal space structure of a square lattice modulated by a longitudinal, displacive modulation along $x$ and $y$, respectively. (**C,D**) Reciprocal space structure of a square lattice modulated by a transverse, displacive modulation along $x$ and $y$, respectively. STEM Fourier transforms and diffraction of BSCMO indicate transverse PLDs.

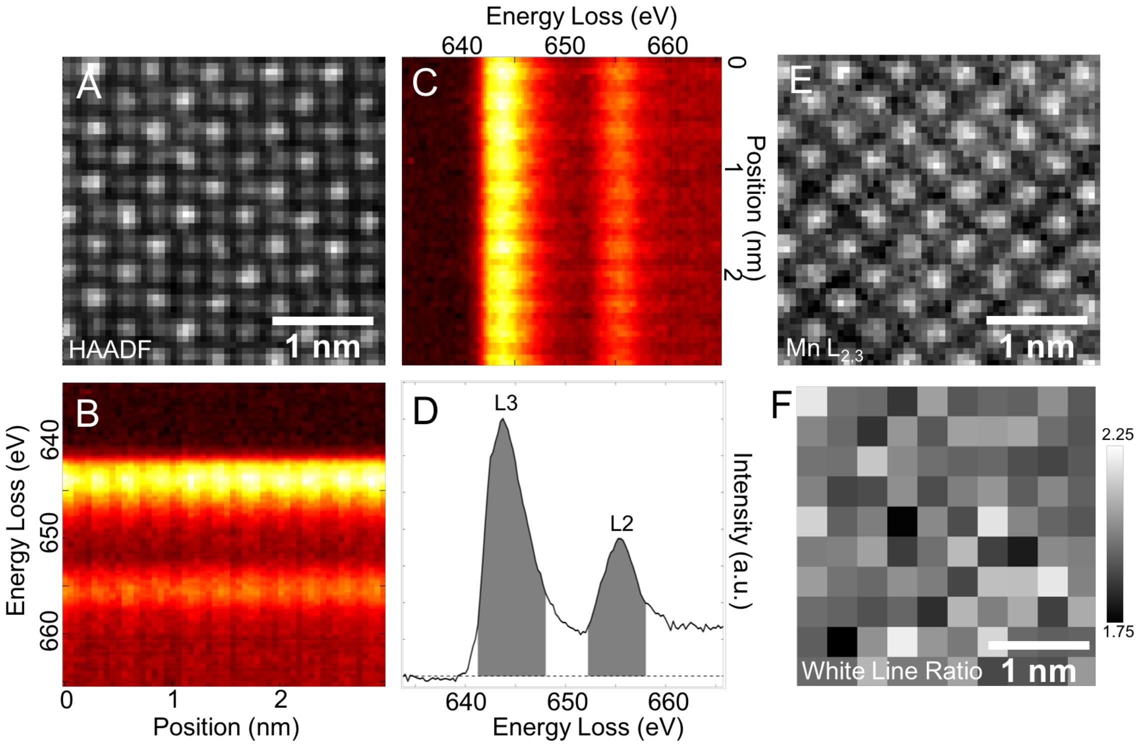


**Supplementary Figure 6: Spectroscopic mapping shows minimal Mn valence changes.** Loudon et al (*13*) placed an upper bound of $\pm 0.04$ on any valence changes in the Mn ions in a compositional variant of BSCMO ($x = 0.5$, $y = 0.1$) using electron energy loss spectroscopy (EELS). Here, we performed aberration-corrected spectroscopic mapping, collecting a full EELS spectrum at each scanned beam position with a pixel dwell time of 0.05 s and and energy dispersion of 0.25 eV. (A) A simultaneously recorded HAADF image shows atomic resolution and little sample drift. Background subtracted EELS spectra integrated along the (B) horizontal and (C) vertical directions show two peaks at ∼640 eV corresponding to the Mn $L_3$ and $L_2$ edges (D). (E) Integrating over both $L_{2,3}$ peaks yields a map of the Mn distribution. Following Loudon et al., we calculated the white line ratio, the ratio of the integrated intensities of $L_3$ to $L_2$ (*gray regions*, D), which tracks valence change. (F) Mapping the white line ratio shows no structure or periodicity above the noise level, consistent with prior work. Note that for (F) the EELS map has been rebinned to the lattice periodicity to improve the SNR.

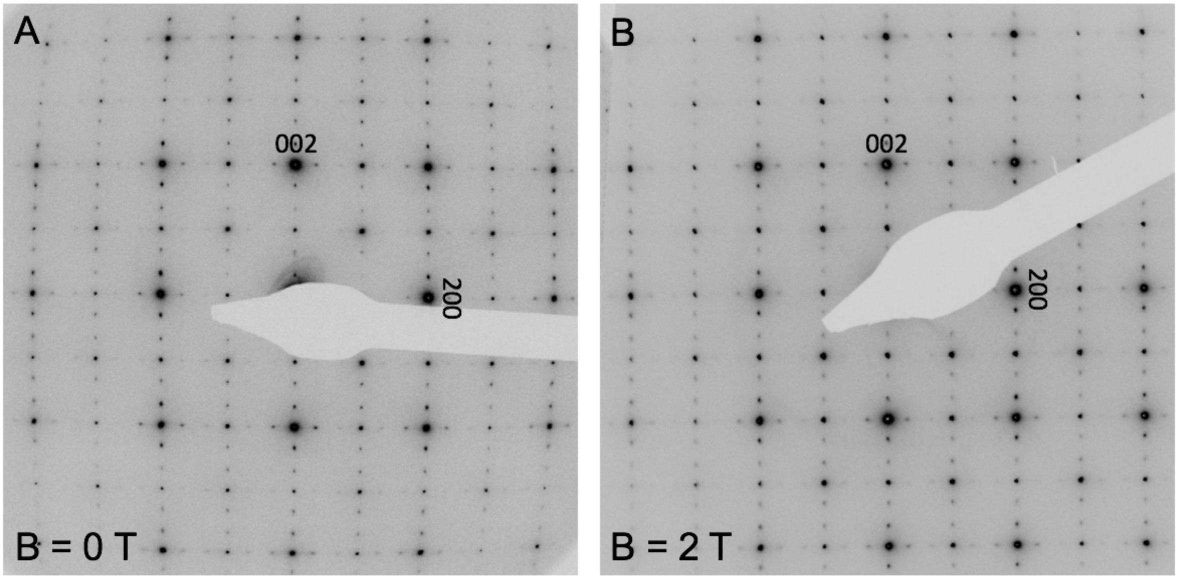


**Supplementary Figure 7: Electron diffraction at 0 T and 2 T.** Electron diffraction in the presence of an applied magnetic field of (A) ∼0 T and (B) ∼2 T. The latter measurement is representative of the sample conditions during STEM imaging, during which the objective lens of the electron microscope induces a strong magnetic field perpendicular to the sample plane. The former measurement was performed with the objective lens turned off, under an estimated magnetic field of $< 3$ mT. Note that the slight bending of the diffraction pattern in (A) results from the imperfect electron optics in field-free conditions. The two diffraction patterns are otherwise comparable, further indicating that the PLDs under study are robust to the object lens' magnetic field.

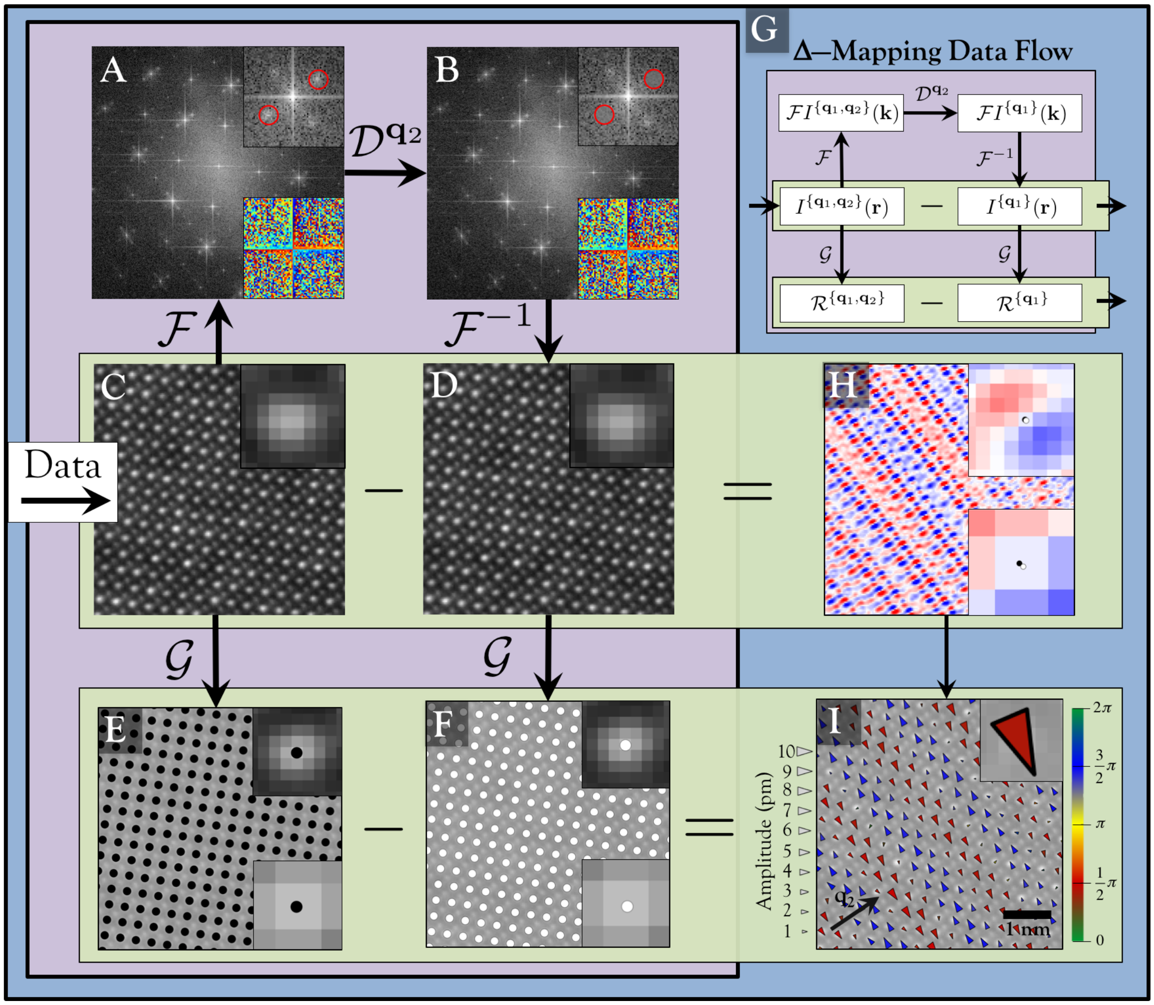


**Supplementary Figure 8: PLD map data processing.** PLD maps are generated beginning with a single high resolution STEM image, obtained by cross correlating and averaging ∼20-30 fast scanned images (C). A Fourier transform is calculated (A), and all satellite peaks corresponding to a modulation wavevector of interest are extracted (*upper inset*). These satellite peaks are damped to the background level (B, *upper inset*) while the phase is left unchanged (A,B, *lower insets*). Taking an inverse Fourier transform yields a reference lattice (D). The difference between the original and reference image qualitatively depict the PLD structure (H). All atomic lattice positions are extracted using 2D Gaussian fits for both the original and calculated reference lattice (E,F). The difference between the fit positions at each lattice site yields the PLD displacements (I). The complete data flow is summarized in (G).

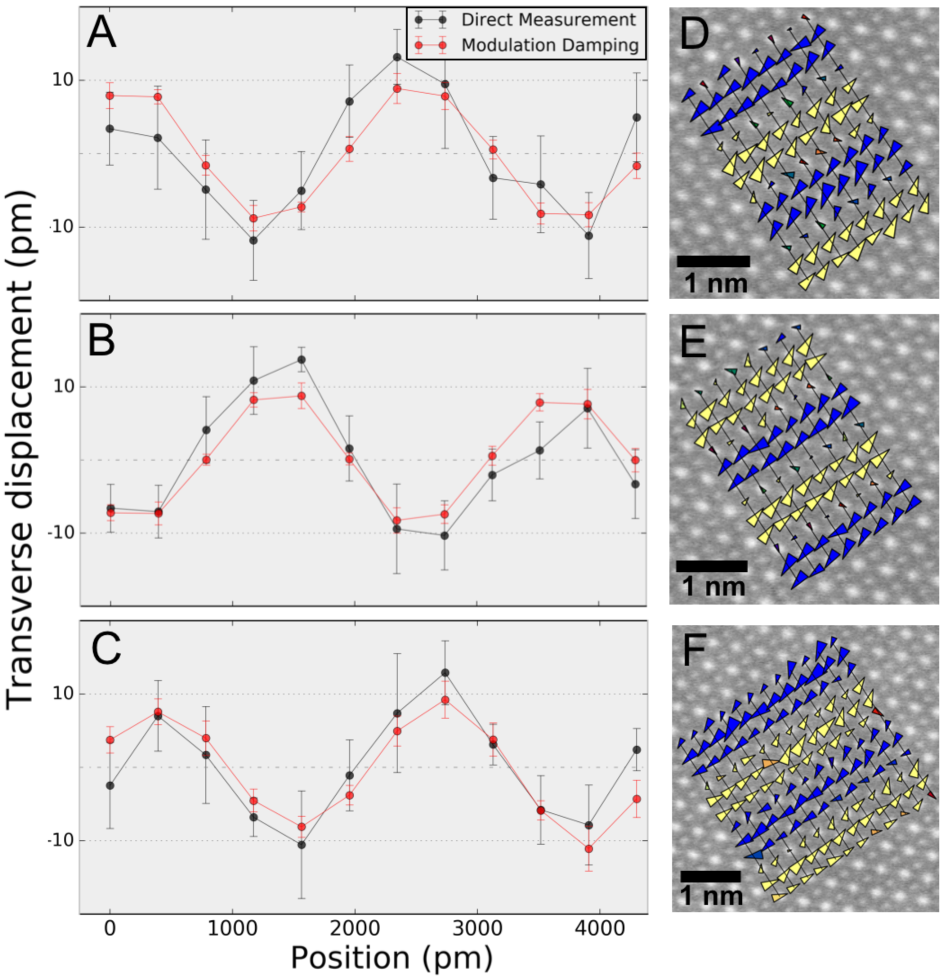


**Supplementary Figure 9: Comparison to PLD measurement without Fourier damping.** Line profiles (A-C) of the transverse component of the displacements are calculated using both the modulation damping approach (*red*) and by defining a local reference line in real space, without any Fourier space modifications (*black*). Circles and error bars represent the mean and standard deviations, respectively, of the transverse displacement measurements across a single row of 8-12 lattice sites in three well-ordered regions (D-F). The two approaches yield consistent results. We believe the larger error bars of the real-space approach primarily reflect that, unlike the modulation damping approach, this method does not account for image distortions, as well as the relative imprecision of using locally defined lines as reference positions.

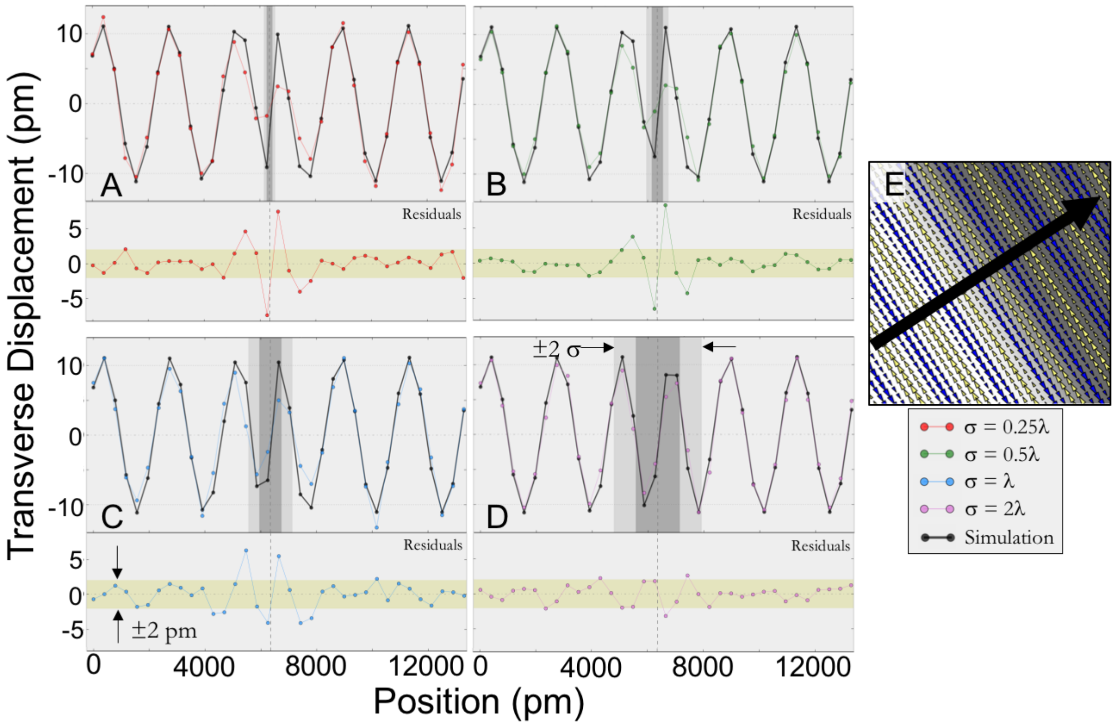


**Supplementary Figure 10: Effect of abrupt PLD features.** Simulated data with antiphase domains in the PLD phase were generated with varying domain boundary width, by blurring a step function phase field with Gaussian kernels given by $\sigma = 0.25\lambda$, $\sigma = 0.5\lambda$, $\sigma = \lambda$, and $\sigma = 2\lambda$ (A-D). Line profiles (E) of the transverse displacement components are plotted for the simulated (*black*) and calculated (*color*) PLDs (A-D, *above*), along with their corresponding residuals (A-D, *below*). Dark/light gray boxes in A-D indicate $\pm\sigma/\pm 2\sigma$ about the domain boundary. Dark/light regions in E indicate PLD phase values of $0/\pi$, with the $\sigma = 2\lambda$ case shown here. The PLDs are accurately captured everywhere for $\sigma = 2\lambda$. The remaining cases all capture the PLDs accurately far from the domain boundaries. For $\sigma = \lambda$ the phase jump is correctly captured qualitatively, but incorrectly damps the displacement amplitudes near the boundary. For $\sigma = 0.25\lambda$ and $\sigma = 0.5\lambda$ the method fails at the atomic sites on the boundary.

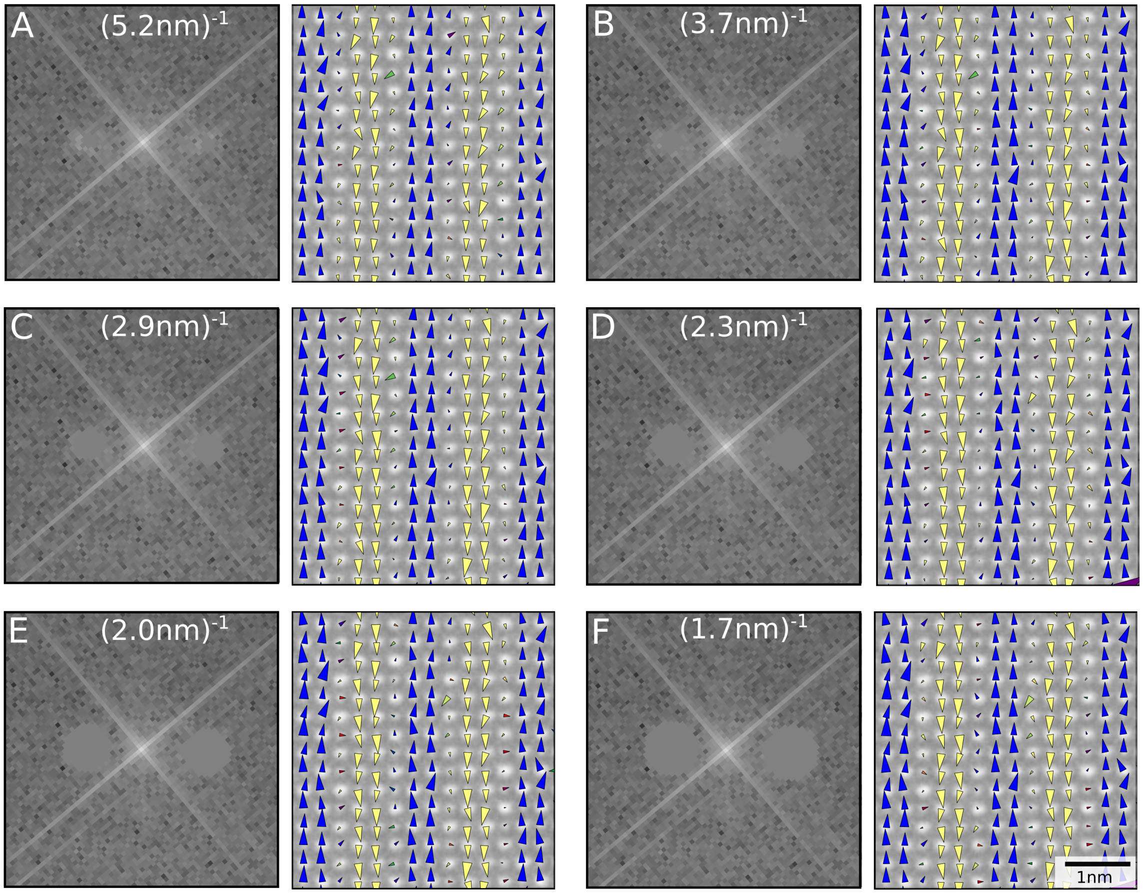


**Supplementary Figure 11: Effect of varying mask size on real data.** (**A-F**) Typical Fourier peaks with reciprocal space mask diameter varying from $(5.2\ \mathrm{nm})^{-1}$ to $(1.7\ \mathrm{nm})^{-1}$ and their respective PLD maps. When the mask size is too small (A), the amplitude of the PLD is diminished since the mask does not reflect the total intensity of the satellite peak. Once the peak is fully captured by the mask, the mapping is insensitive to increasing mask size.

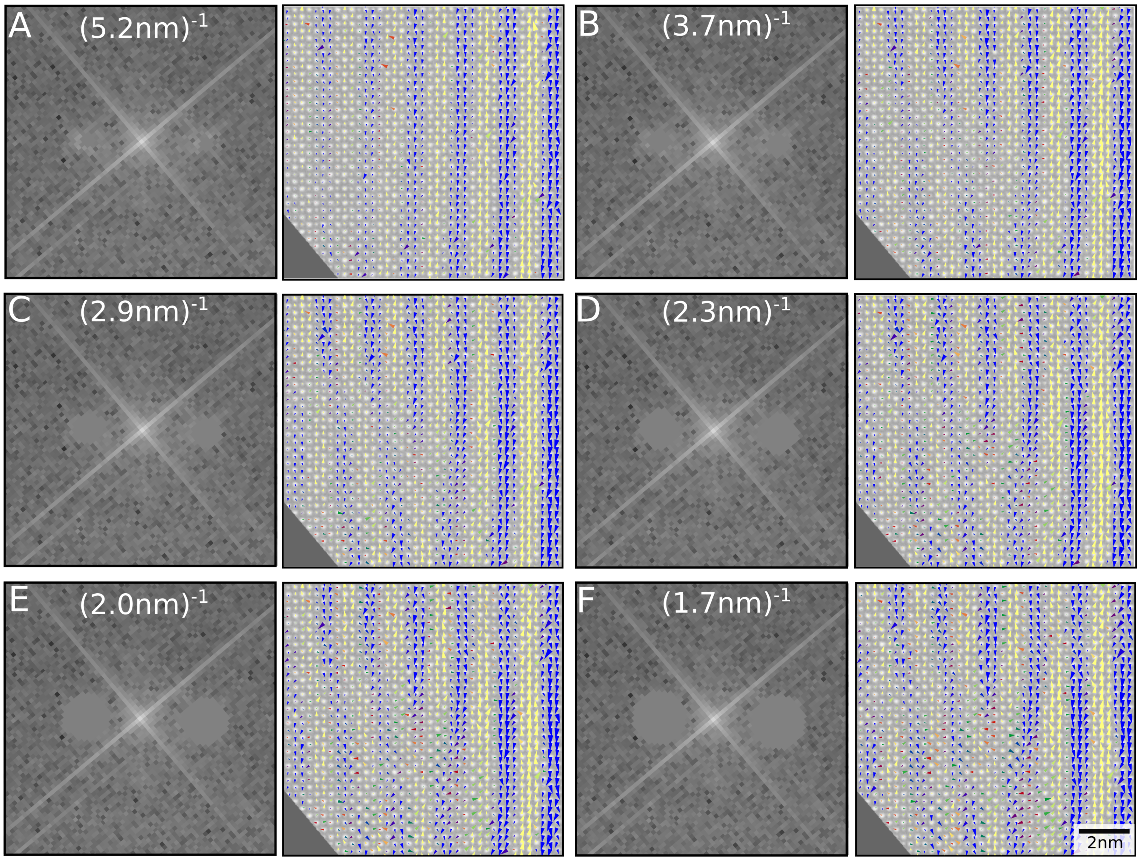


**Supplementary Figure 12: Effect of varying mask size on mapping of a topological defect.** (**A-F**) Typical Fourier peaks with reciprocal space mask diameter varying from $(5.2\ \mathrm{nm})^{-1}$ to $(1.7\ \mathrm{nm})^{-1}$ and the respective PLD maps of a topological defect. The topological defect is missing in A because the mask does not fully capture the satellite peak. For mask sizes that fully capture by the satellite peaks, the mapping is insensitive to increasing mask size.

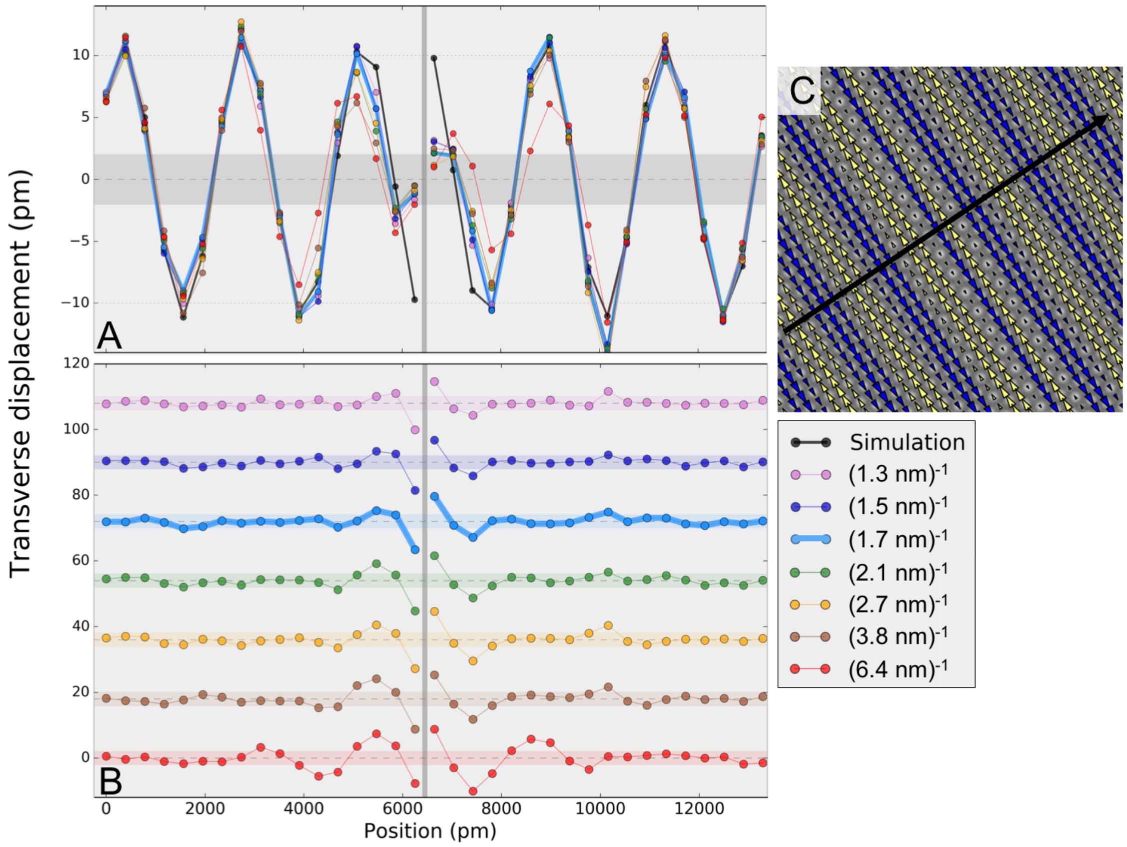


**Supplementary Figure 13: Effect of varying mask size on simulated data.** Simulated data with a Heavyside function antiphase domain boundary in the PLD phase was generated, and the PLD reconstructed using 7 different mask sizes. Line profiles of the transverse displacement components are plotted for the simulated (*black*) and calculated (*color*) PLDs (A), along with their corresponding residuals (B), for the sites indicated in the PLD map (C). The horizontal bars in A (*gray*) and B (*colors*) indicate $\pm 2$ pm. All mask sizes accurately capture the displacements far from the interface, and fail to capture the true displacements of the two sites at the atomically sharp antiphase interface. Very small Fourier space masks (*red, brown*) result in artifacts in the residuals several lattice spacings or more from the interface. For large Fourier space masks (*light blue, dark blue, purple*) the residuals are on the order of the $\pm 2$ pm error bars 2 lattice spacings from the interface. The typical mask size used on experimental data is bolded (*light blue*, $(1.7\text{ nm})^{-1}$.

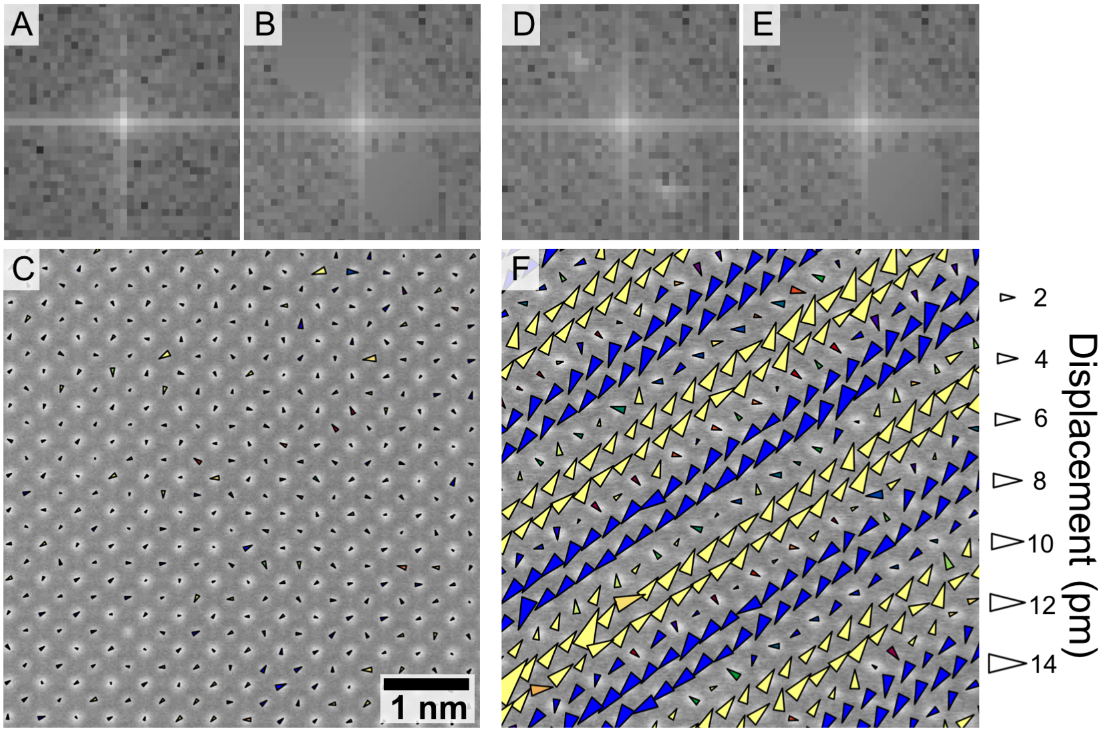


**Supplementary Figure 14: PLD mapping in STO vs BSCMO** The Fourier transform of $SrTiO_3$ (STO) data does not contain satellite peaks about the Bragg peaks (A), while the Fourier transform of BSCMO data does (D). The damping procedure was performed on both STO data (B) and BSCMO data (E) at comparable positions in Fourier space. The resulting PLD map for STO data shows displacement vectors with a mean magnitude of 0.390 pm and no clear periodic structure (C), in stark contrast to the strong PLD structure observed for BSCMO PLD map (F). The image and displacement vector size scales are identical for (C) and (F).

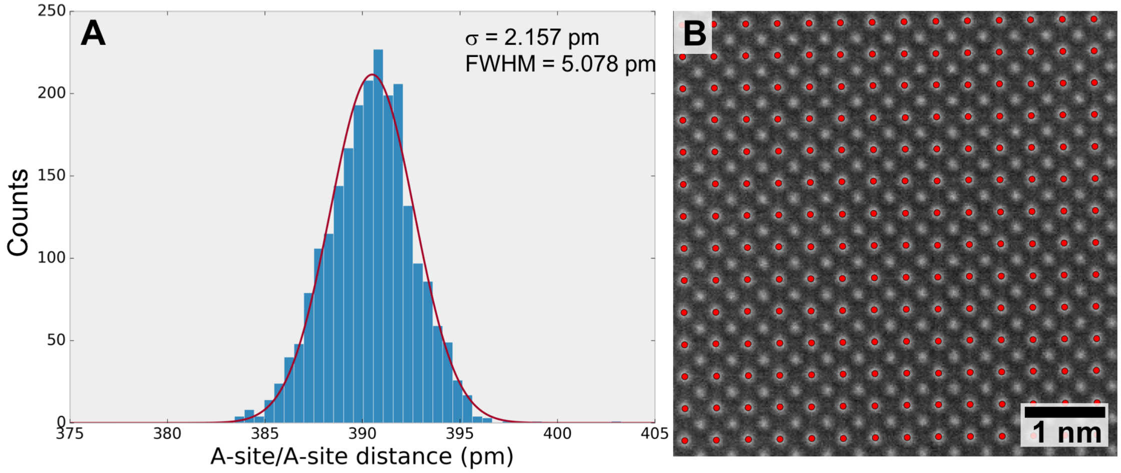


**Supplementary Figure 15: Precision of atomic position fits (A)** A histogram of distances between neighboring Sr sites in STO data **(B)** is used to estimate the precision of the atomic positions extracted via 2D Gaussian fits. A 1D Gaussian fit to the resulting histogram has $\sigma = 2.157$ pm and FWHM$= 5.078$ pm. Identical analysis for the lower signal-to-noise ratio Ti sites yields $\sigma = 2.415$ pm and FWHM$= 5.687$ pm.

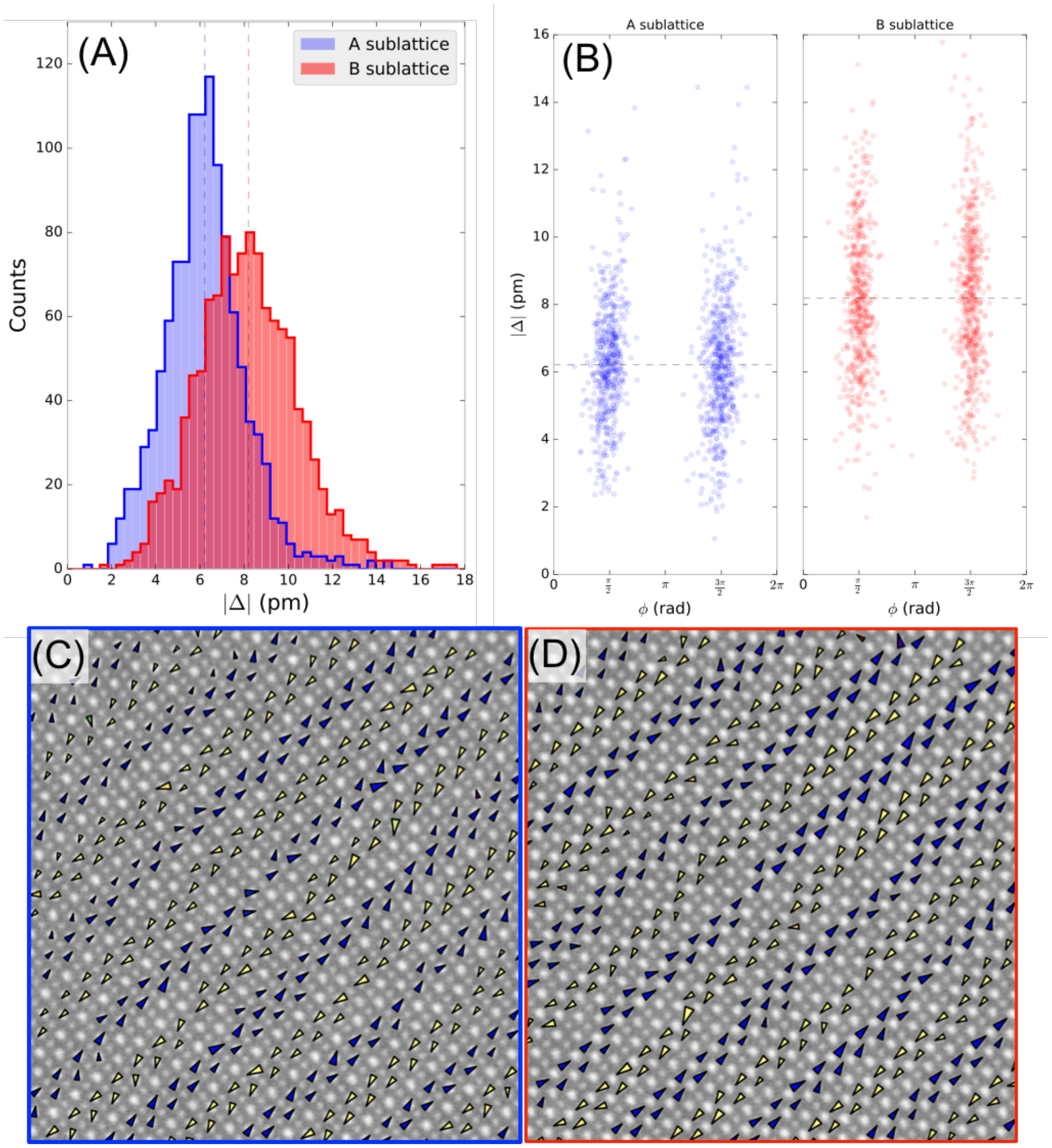


**Supplementary Figure 16: A and B sublattice amplitudes** (**A**) Histograms of the displacement magnitudes at the PLD maxima in well-ordered regions on the A-sublattice (*blue*) and B-sublattice (*red*). The mean magnitudes at these sites is 6.2 pm / 8.2 pm on the A- / B-sublattices, respectively (*dashed lines*). (**B**) Scatterplots of the PLD displacement magnitude versus polarization angle $\phi$ for the A- and B-sublattices. The dashed lines again show the mean displacements at the sites analyzed. (**C,D**) Sample sites used in analyzing the displacement magnitudes on the A- and B-sublattice are shown in C and D, respectively. To extract meaningful values we examined only sites corresponding to local PLD maxima.

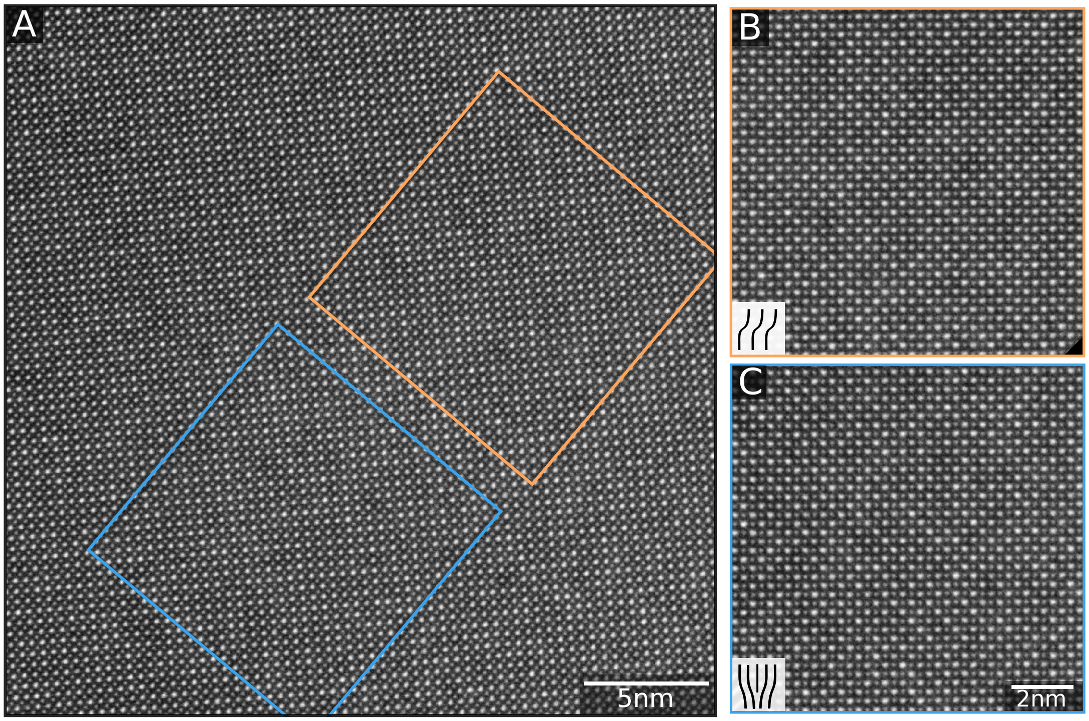


**Supplementary Figure 17: HAADF lattice image of BSCMO.** (**A**) Original, unrotated HAADF image corresponding to results in main text. (**B**) Raw data corresponding to the region containing the shear deformation shown in Figs. 4A-C in the main text. (**C**) Raw data corresponding to the region containing the dislocation shown in Figs. 4D-F in the main text. No dislocations are observed in the underlying lattice, supporting that observed PLD defects in main text are intrinsic to the modulations. HAADF data are unprocessed except for registration and alignment of image series (see *Methods* in the main text). The A-sites exhibit varying intensities indicating quenched impurity disorder due to Bi/Sr/Ca doping.

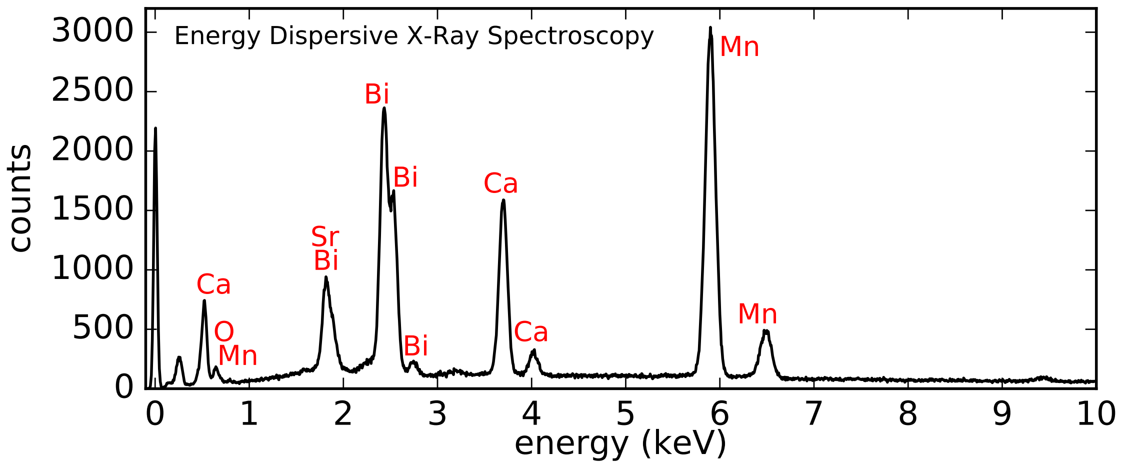


**Supplementary Figure 18: Energy Dispersive X-ray Spectroscopy** The sample composition was determined using energy dispersive X-ray spectroscopy. We determined the $Bi_{1-x}Sr_{x-y}Ca_yMnO_3$ composition to be approximately $x = 0.65$ and $y = 0.47$. We observed negligible variations in the composition across the sample.

# Supplementary Notes

## Supplementary Note 1: Periodic Lattice Displacement Mapping Method

### *Overview*

Extracting local atomic positions with picometer precision is a well established and powerful tool for analysis of high resolution scanning transmission electron microscopy (STEM) data, and has been used to great effect in describing, for example, local polarization in ferroelectrics, interfacial coupling in oxide heterostructures, and tunable octahedral rotations (*1–8*). However, the ability to measure the positions of atomic nuclei is a necessary but not sufficient condition to locally map periodic lattice displacements. The key challenge is defining a reference lattice: in order to calculate the atomic displacements, each atomic position measured in the raw data must be compared to some suitable reference position. In ferroelectrics, defining a reference is comparatively straightforward, for example by measuring the displacements of a central B-site atom with respect to a surrounding A-site cage or an oxygen cage in a unit cell of $ABO_3$ (*1–5*). Here, in contrast, there exists no simple reference against which to measure the displacement of a given atomic site, further exacerbated by the possibility of disorder, distinct sublattice behavior, and multiple modulation wavevectors.

The method used in this report defines a reference lattice against which the displacements of interest can be measured by leveraging the convenient decoupling of the periodic lattice displacement (PLD) from its underlying unmodulated lattice in Fourier space: the unmodulated lattice appears in Fourier space as the usual Bragg peaks, and the PLD as satellite peaks decorating each Bragg peak. By carefully damping the satellite peaks in Fourier space, the contribution of the PLD only is removed, and a reference lattice can be extracted. In addition to making local PLD mapping tenable in the first place, this approach has two notable advantages: first, multiple independent modulations can be individually extracted and mapped because they are decoupled in Fourier space; and second, local distortions from the imaging process are naturally accounted for because they are present in both the original and reference images.

Below, the details of the method are described. First, the data processing procedure itself is presented. Next, the validity of the approach is confirmed by comparison to lattice displacements calculated using a coarser approach which involves only real space measurements from the raw data. The limits and regime of validity of the approach are then discussed and illustrated using a variety of simulated datasets, and rigorous interpretation of the extracted displacements is addressed. The importance of judiciously choosing an appropriate Fourier space mask is then discussed. Finally, we discuss important analytical details of our approach relating PLDs in real space and Fourier space.

### *Data Processing*

The algorithm used to produce periodic lattice displacement maps is summarized in Supplementary Figure 8, and begins with a single high resolution STEM image (Supplementary Fig-

ure 8c). Let $I(\mathbf{r})$ be the input STEM image, where $\mathbf{r} \in \mathbb{R}^2$, let $\mathcal{R}$ be the set of all atomic column positions $\mathbf{R}$, and let $\delta(\mathbf{r})$ be the Dirac delta function. We write the image $I(\mathbf{r})$ as

$$I(\mathbf{r}) = \sum_{\mathbf{R}\in\mathcal{R}} f\left(\mathbf{r} - \mathbf{R}\right) \tag{1}$$

$$= f\left(\mathbf{r}\right) * \sum_{\mathbf{R}\in\mathcal{R}} \delta\left(\mathbf{r} - \mathbf{R}\right) \tag{2}$$

where $*$ indicates a convolution and $f(\mathbf{r})$ is a form factor describing the STEM signal about each atomic site, incorporating the scattering cross section of high energy electrons with the projected potentials of the atomic columns, the finite point spread function of the electron beam, and channeling effects. For simplicity we consider the case of a single atomic species here, however, it is possible to include multiple form factors $f_i(\mathbf{r})$.

Atomic columns in STEM images are fit to two dimensional Gaussian functions and their positions extracted (Supplementary Figure 8e). This process can be considered a transformation which accepts an image $I(\mathbf{r})$ of the form in Supplementary Equation (1) and outputs the set of all atomic positions in the image, $\mathcal{R}$. That is

$$\mathcal{G}\left(I(\mathbf{r})\right) = \mathcal{G}\left(f\left(\mathbf{r}\right) * \sum_{\mathbf{R}\in\mathcal{R}} \delta\left(\mathbf{r} - \mathbf{R}\right)\right) \equiv \mathcal{R} \tag{3}$$

Above, the set $\mathcal{R}$ is generic; let $\mathcal{R}^{\{0\}}$ be the set of all lattice points in an unmodulated lattice, which we here take for simplicity to be a Bravais lattice in two dimensions, $\mathcal{R}^{\{0\}} \equiv \{\mathbf{R}_{ij}^{\{0\}} = i\mathbf{a}_1 + j\mathbf{a}_2 \mid i, j \in \mathbb{Z}\}$. For a lattice with a single sinusoidal modulation given by a modulation wavevector $\mathbf{q}_1$, we then write the set of all lattice points as $\mathcal{R}^{\{\mathbf{q}_1\}}$, for a lattice with two coexisting modulations $\mathbf{q}_1$ and $\mathbf{q}_2$ we write $\mathcal{R}^{\{\mathbf{q}_1,\mathbf{q}_2\}}$, and for a general set of modulation wavevectors $\mathcal{Q} \equiv \{\mathbf{q}_i \mid i \in 1 \ldots N\}$, we write $\mathcal{R}^{\mathcal{Q}}$ to indicate the set of all lattice points in the lattice modulated by all $\mathbf{q} \in \mathcal{Q}$. Then $\mathcal{R}^{\mathcal{Q}} \equiv \{\mathbf{R}_{ij}^{\mathcal{Q}} \mid i, j \in \mathbb{Z}\}$, where modulated lattice sites may now be written in terms of the unmodulated lattice sites as

$$\mathbf{R}_{ij}^{\mathbf{q}} = \mathbf{R}_{ij}^{\{0\}} + \mathbf{A}\sin\left(\mathbf{q}\cdot\mathbf{R}_{ij}^{\{0\}} + \phi\right) \tag{4}$$

$$\mathbf{R}_{ij}^{\mathcal{Q}} = \mathbf{R}_{ij}^{\{0\}} + \sum_{\mathbf{q}\in\mathcal{Q}} \mathbf{A}_{\mathbf{q}}\sin\left(\mathbf{q}\cdot\mathbf{R}_{ij}^{\{0\}} + \phi_{\mathbf{q}}\right) \tag{5}$$

Here we focus on the case of sinusoidal modulations, but periodic modulations with more general waveforms are implicitly included by allowing $\mathcal{Q}$ to include higher order Fourier components.

The displacement of atomic column $(i, j)$ for a lattice with a single modulation vector $\mathbf{q}_1$ can then be written as $\mathbf{\Delta}_{ij}^{\mathbf{q}_1} = \mathbf{R}_{ij}^{\mathbf{q}_1} - \mathbf{R}_{ij}^{\{0\}}$. More generally, for a lattice with multiple coexisting modulations $\mathcal{Q}$, the displacement of each atomic column resulting solely from modulation vector $\mathbf{q}_p$ is

$$\mathbf{\Delta}_{ij}^{\mathbf{q}_p} = \mathbf{R}_{ij}^{\mathcal{Q}} - \mathbf{R}_{ij}^{\mathcal{Q}\setminus\mathbf{q}_p} \tag{6}$$

where $\mathcal{Q}\backslash\mathbf{q}_p$ indicates the set $\mathcal{Q}$ with element $\mathbf{q}_p$ removed. Thus, Supplementary Equation (6) simply defines the displacement at each atomic column due to a single modulation as the difference between atomic positions with and without that modulation present.

Once the lattice positions $\mathbf{R}_{ij}^{\mathcal{Q}}$ have been extracted by fitting Gaussians to each atomic site from an experimental image $I(\mathbf{r}) = I^{\mathcal{Q}}(\mathbf{r})$, via $\mathcal{G}\left(I^{\mathcal{Q}}(\mathbf{r})\right) = \mathcal{R}^{\mathcal{Q}}$, all that remains is to find the positions of a reference lattice in which the single modulation vector of interest $\mathbf{q}_p$ has been removed, $\mathcal{R}^{\mathcal{Q}\backslash\mathbf{q}_p}$. This may be obtained by fitting the atomic columns of an image $I^{\mathcal{Q}\backslash\mathbf{q}_p}(\mathbf{r})$ in which $\mathbf{q}_p$ has been removed (Supplementary Figure 8d), because $\mathcal{G}\left(I^{\mathcal{Q}\backslash\mathbf{q}_p}(\mathbf{r})\right) = \mathcal{R}^{\mathcal{Q}\backslash\mathbf{q}_p}$ (Supplementary Figure 8f). The task is therefore to obtain image $I^{\mathcal{Q}\backslash\mathbf{q}_p}(\mathbf{r})$ from an experimental image $I^{\mathcal{Q}}(\mathbf{r})$.

Removing the contribution of a single modulation is most naturally approached in Fourier space. Let $\mathcal{F}I(\mathbf{k}) = \int I(\mathbf{r})e^{-i\mathbf{k}\cdot\mathbf{r}}d\mathbf{r}$ be the Fourier transform of an image $I(\mathbf{r})$. For an image $I^{\mathcal{Q}}(\mathbf{r})$ defined according to Supplementary Equations (1,5), the Fourier transform may be written as

$$\begin{aligned}\mathcal{F}I^{\mathcal{Q}}(\mathbf{k}) =& \mathcal{F}f(\mathbf{k}) \\ & \cdot \sum_{\alpha_{\mathbf{q}_1}=-\infty}^{\infty} \cdots \sum_{\alpha_{\mathbf{q}_N}=-\infty}^{\infty} \sum_{\mathbf{b}\in\mathcal{B}^{\{0\}}} \delta\left(\mathbf{k} - \left(\mathbf{b} - \sum_{\mathbf{q}\in\mathcal{Q}} \alpha_{\mathbf{q}}\mathbf{q}\right)\right) \\ & \cdot \prod_{\mathbf{q}\in\mathcal{Q}} \mathcal{M}_{\alpha_{\mathbf{q}},\mathbf{q}}(\mathbf{k}) \end{aligned} \tag{7}$$

where $\mathcal{B}^{\{0\}}$ is the reciprocal lattice of $\mathcal{R}^{\{0\}}$, and

$$\mathcal{M}_{\alpha_{\mathbf{q}},\mathbf{q}}(\mathbf{k}) \equiv J_{\alpha_{\mathbf{q}}}\left(\mathbf{k}\cdot\mathbf{A}_{\mathbf{q}}\right)\exp\left[i\alpha_{\mathbf{q}}\phi_{\mathbf{q}}\right] \tag{8}$$

where $J_\alpha(z)$ is the $\alpha$'th Bessel function of the first kind. Derivation of Supplementary Equation 7 is found at the end of this section, and related calculations are found in (*9, 10*). Here, the important observation is that for each of the Bragg peaks on the reciprocal lattice sites, $\delta\left(\mathbf{k}-\mathbf{b}\right)$ for $\mathbf{b}\in\mathcal{B}^{\{0\}}$, there are an additional set of satellite peaks offset from the Bragg peak by the linear combinations of the modulation vectors $\mathbf{q}\in\mathcal{Q}$, which encode the PLD. The PLD is thus effectively decoupled from the underlying lattice in Fourier space.

In the experimental BSCMO STEM data here, only first order peaks for the two modulation vectors $\mathbf{q}_1$, $\mathbf{q}_2$ are observed, due to the damping of the higher order harmonics according to $|\mathcal{M}_\alpha(\mathbf{k})| \approx \frac{1}{\alpha}\left(\frac{1}{2}\mathbf{k}\cdot\mathbf{A}_{\mathbf{q}}\right)^\alpha$, where we've used the fact that the argument $\mathbf{k}\cdot\mathbf{A}_{\mathbf{q}} \ll 1$ (here, $|\mathbf{A}_{\mathbf{q}}| \approx 10$ pm and $\mathbf{k} \leq (1\text{Å})^{-1}$, thus $\mathbf{k}\cdot\mathbf{A}_{\mathbf{q}} \leq 0.1$). Including only the experimentally observable peaks reduces Supplementary Equation 7 to

$$\begin{aligned}\mathcal{F}I^{\mathbf{q}_1,\mathbf{q}_2}(\mathbf{k}) = \mathcal{F}f(\mathbf{k}) \sum_{\mathbf{b}\in\mathcal{B}^{\{0\}}} & c_0\delta\left(\mathbf{k}-\mathbf{b}\right) + c_{-,1}\delta\left(\mathbf{k}-\left(\mathbf{b}-\mathbf{q}_1\right)\right) + c_{+,1}\delta\left(\mathbf{k}-\left(\mathbf{b}+\mathbf{q}_1\right)\right) \\ & + c_{-,2}\delta\left(\mathbf{k}-\left(\mathbf{b}-\mathbf{q}_2\right)\right) + c_{+,2}\delta\left(\mathbf{k}-\left(\mathbf{b}+\mathbf{q}_2\right)\right)\end{aligned} \tag{9}$$

for complex constants $c$.

We then define a transformation $\mathcal{D}^{\mathbf{q}_p}$ which removes the contribution of modulation $\mathbf{q}_p$ from a Fourier transform. By definition

$$\mathcal{D}^{\mathbf{q}_p}\left(\mathcal{F}I^{\mathcal{Q}}(\mathbf{k})\right) \equiv \mathcal{F}I^{\mathcal{Q}\setminus\mathbf{q}_p}(\mathbf{k}) \tag{10}$$

Specifically

$$\begin{aligned}\mathcal{D}^{\mathbf{q}_1}\left(\mathcal{F}I^{\mathbf{q}_1,\mathbf{q}_2}(\mathbf{k})\right) = \mathcal{F}f(\mathbf{k}) \sum_{\mathbf{b}\in\mathcal{B}^{\{0\}}} & c_0\delta\left(\mathbf{k}-\mathbf{b}\right) \\ & + c_{-,2}\delta\left(\mathbf{k}-(\mathbf{b}-\mathbf{q}_2)\right) + c_{+,2}\delta\left(\mathbf{k}-(\mathbf{b}+\mathbf{q}_2)\right) \qquad (11)\\ \mathcal{D}^{\mathbf{q}_2}\left(\mathcal{F}I^{\mathbf{q}_1,\mathbf{q}_2}(\mathbf{k})\right) = \mathcal{F}f(\mathbf{k}) \sum_{\mathbf{b}\in\mathcal{B}^{\{0\}}} & c_0\delta\left(\mathbf{k}-\mathbf{b}\right) \\ & + c_{-,1}\delta\left(\mathbf{k}-(\mathbf{b}-\mathbf{q}_1)\right) + c_{+,1}\delta\left(\mathbf{k}-(\mathbf{b}+\mathbf{q}_1)\right) \qquad (12)\end{aligned}$$

Obtaining $\mathcal{D}^{\mathbf{q}_1}$ and $\mathcal{D}^{\mathbf{q}_2}$ thus requires carefully removing the relevant peaks from the experimental Fourier transform (Supplementary Figure 8a). Algorithmically, the positions of all detectable satellite peaks corresponding to a single modulation wavevector $\mathbf{q}$ of interest are extracted (Supplementary Figure 8a, upper inset). A mask radius is chosen, and the background level for each satellite peak is calculated by finding a 2D linear fit to the Fourier space amplitude in an annulus about that mask. The amplitudes inside each masked region is then scaled down to this background level (Supplementary Figure 8b, upper inset), while leaving the phase information unaltered (Supplementary Figure 8a,b, lower insets), yielding $\mathcal{D}^{\mathbf{q}_p}$.

An inverse Fourier transform is taken to obtain a $\mathbf{q}$–damped reference image,

$$\mathcal{F}^{-1}\left(\mathcal{D}^{\mathbf{q}_p}\left(\mathcal{F}\left(I^{\mathcal{Q}}(\mathbf{r})\right)\right)\right) = I^{\mathcal{Q}\setminus\mathbf{q}_p}(\mathbf{r}) \tag{13}$$

where $\mathcal{F}^{-1}$ is the inverse Fourier transform (Supplementary Figure 8d). The positions of all atomic sites in $I^{\mathcal{Q}\setminus\mathbf{q}_p}(\mathbf{r})$ are then extracted by fitting Gaussians to each site (Supplementary Figure 8f), i.e.

$$\mathcal{G}\left(\mathcal{F}^{-1}\left(\mathcal{D}^{\mathbf{q}_p}\left(\mathcal{F}\left(I^{\mathcal{Q}}(\mathbf{r})\right)\right)\right)\right) = \mathcal{R}^{\mathcal{Q}\setminus\mathbf{q}_p} \tag{14}$$

With both sets of atomic positions $\mathcal{R}^{\mathcal{Q}}$ and $\mathcal{R}^{\mathcal{Q}\setminus\mathbf{q}_p}$ in hand, $\Delta_{i,j}^{\mathbf{q}_p}(\mathbf{r})$ may then be directly calculate via Supplementary Equation 6 (Supplementary Figure 8i). A qualitative picture of the PLD structure may be obtained sans Gaussian fits by taking $I^{\mathcal{Q}}(\mathbf{r}) - I^{\mathcal{Q}\setminus\mathbf{q}_p}(\mathbf{r})$, shown in Supplementary Figure 8h. The complete data processing flow is summarized in Supplementary Figure 8g.

As with any processing performed on raw data, in order to correctly interpret the results of this approach it is necessary to carefully understand precisely its limits, regime of validity, and any possible artifacts. The sections below discuss these points through a combination of simulation, experimental control datasets, and theoretical considerations.

*Comparison with Direct, Real Space PLD Measurement*

In order to confirm that the measured PLDs are not an artifact of the Fourier space damping procedure, we calculated the displacement vectors directly from the data, unprocessed except for cross correlation, in real space. In Supplementary Figure 9, the transverse component of the displacement vectors obtained using the modulation damping approach (red) are compared to the transverse distances of atomic centers from a line fit to the positions of all centers (black) along a column in the q-vector direction (lines, D-F) spanning two PLD wavelengths. This real-space approach useful for confirming the validity of the method, however, it is only possible in well-ordered regions containing a single modulation. Three different well-ordered regions of an experimental dataset, in which a single modulation dominates, are shown. Circles and error bars in the line profiles (Supplementary Figure 9A-C) represent the mean and standard deviations of the transverse displacements along a single row perpendicular to $\mathbf{q}$.

The results are in good agreement on average, clearly indicating that the modulation damping approach is indeed reconstructing a displacement field present in the data. Notably, the real space approach has significantly larger error bars than the Fourier damping approach. We attribute this to three factors. First, the real space approach does not account for distortions resulting from the imaging process, limiting its accuracy. Second, using a best-fit line as a reference position is a somewhat coarse approach, however, more systematic real-space methods such as a global coordinate rotation were untenable, likely due precisely to image distortion. Third, the reference lattice defined by the Fourier space method effectively represents a locally averaged reference structure, thus, sufficiently localized features in the displacement field may be smoothed out. The following sections explore this last possibility in greater detail, and demonstrate that all but the very sharpest features in the PLD field are well described by the Fourier damping approach.

*Method Limits, Regime of Validity, and Interpretation*

For a perfect lattice modulated by a perfect sinusoidal displacement field, both the Bragg and satellite peaks are delta functions. Local disorder in the PLD field causes the satellite peaks to deviate from perfect impulses. The size scale of the local features in the PLD structure relate to the degree of blurring observed in the satellite peaks, therefore the size of the damping mask used (Supplementary Figure 8a,b *upper insets*) determines the sharpness of the PLD features the method is able to reconstruct with good fidelity. To capture the highest frequency variations in the PLD field possible, the largest mask size which does not interfere with other Fourier space features should be chosen. In this work we used masks that extended halfway to the nearest Bragg peaks. The mask radius is therefore approximately $|\frac{1}{2}\mathbf{q}|$, and we expect to correctly capture any PLD disorder features of size $\gtrsim 2\lambda_{\text{PLD}}$. For PLD disorder of size scales $\lesssim 2\lambda_{\text{PLD}}$, our reconstructed displacements may deviate from the true displacement magnitudes somewhat. The simulations discussed below demonstrate that this effect is only appreciable at atomically sharp disorder in the PLD field, and we believe our reconstructed displacements are correct everywhere, with the possible exception of the atomic sites located precisely at topological

defect cores.

To carefully understand these effects and ensure correct interpretation of our results, we simulated data with a single sinusoidal modulation, and an antiphase domain boundary separating regions in which the PLD phase shifts by $\pi$. Four datasets were simulated, varying the abruptness of the antiphase domain boundary by first generating a step function domain boundary and then blurring with a Gaussian kernel with four different values of $\sigma$. Supplementary Figure 10 A-D show the results for $\sigma = 0.25\lambda_{\mathrm{PLD}}$, $\sigma = 0.5\lambda_{\mathrm{PLD}}$, $\sigma = \lambda_{\mathrm{PLD}}$, and $\sigma = 2\lambda_{\mathrm{PLD}}$, respectively. Each shows the simulated and calculated transverse displacement components along the line profile shown in Supplementary Figure 10E, along with their residuals. In every case, the residuals fall inside our estimated $\sim \pm 2$ pm precision within a few lattice spacings of the interface. Further, in every case the reconstructed displacements accurately capture the qualitative structure of the antiphase boundary everywhere except for at the lattice sites precisely at the center of the interface. Quantitatively, the reconstructed displacements accurately capture the simulated data everywhere for the $\sigma = 2\lambda_{\mathrm{PLD}}$ case. For smaller $\sigma$, the residuals are somewhat larger within $\sim \lambda_{\mathrm{PLD}}$ about the interface, as expected. For very sharp interfaces ($\sigma = 0.25\lambda_{\mathrm{PLD}}$ and $\sigma = 0.5\lambda_{\mathrm{PLD}}$) the reconstructed displacements are incorrect at the center of the interface, where the atomically sharp discontinuity in the simulated displacement field is averaged out in the reconstruction to yield incorrectly small displacements.

We conclude that for local PLD disorder of size $\gtrsim 2\lambda_{\mathrm{PLD}}$, our approach is valid everywhere. At smaller features, our approach correctly captures qualitative structure but tends to underestimate displacement amplitudes near the feature center. The approach fails entirely only at atomically abrupt features in the PLD field. We contend that this approach therefore reasonably reflects the PLD structure everywhere in the data presented, with the possible exception of the topological defect cores. We believe the reconstructed displacements about defect cores are likely correct, because of smoothing and amplitude damping of the total displacement vectors near phase singularities, however, we cannot discount the possibility that we have averaged over a sharp discontinuity at a defect core. In this scenario, the reconstructed displacements would only be incorrect at the sites directly adjacent to the core, and the amplitudes at these sites may be considered a lower bound on their true displacement amplitudes. Note that we cannot experimentally discount atomically sharp disorder elsewhere in the PLD field, however, we believe such features to be unlikely on energetic grounds.

*Effect of Fourier Mask Size*

We tested the effect of varying the mask size on experimental data, in both a well ordered region and a disordered region containing a topological defect, shown in Figs. 11 and Supplementary Figure 12, respectively. In both cases, a very small mask does not damp the full intensity of the satellite peak. In ordered regions, this leads to artificially reduced PLD amplitude relative to the other PLD maps of the same data (Figs. 11A and 12A). A very large mask begins to introduce greater noise, observable at the modulation minima, and likely resulting from beginning to damp some of the Fourier space intensity in the tails of the nearby Bragg peak. In the intermediate

range of mask sizes which cover the entire satellite peak but remain far from the Bragg peak, the reconstructed PLD is insensitive to mask size variation. We additionally tested the effect of introducing noise into the Fourier space damping level comparable to the noise observed nearby in Fourier space, with negligible effect.

In order to further understand the effect of the Fourier mask size, we varied the mask size used to reconstruct a simulated dataset containing an antiphase domain boundary in the PLD phase. To test how our PLD reconstruction approach behaves in the worst case scenario, in this simulated data the $0$ to $\pi$ transition in the PLD phase is given by a perfect step function. Supplementary Figure 13 shows the results for 7 mask sizes, corresponding to real space diameters of 1.3 nm, 1.5 nm, 1.7 nm, 2.1 nm, 2.7 nm, 3.8 nm, and 6.4 nm. In Supplementary Figure 13 A, the transverse components of the simulated displacements (*black*) agree well with their reconstructed counterparts far from the boundary. The single atomic sites directly adjacent to either side of the boundary are incorrect in all cases, as expected for an atomically sharp feature and discussed in the previous section. The fidelity of the reconstruction in the intermediate region is evident in the residuals, Supplementary Figure 13 B. The vertical scale is indicated by the colored horizontal bars, which correspond to the gray horizontal bar in Supplementary Figure 13 A and represent the $\pm 2$ pm error bars. The smallest Fourier space mask (*red*, 6.4 nm) displays damped periodic ringing in the residuals reminiscent of a sinc function. This artifact is gradually reduced as the mask size is increased (and its corresponding real space distance is decreased), with residuals for the largest masks falling inside the $\pm 2$ pm error bars within 2-3 lattice spacings of the interface. The typical mask size used on experimental data, corresponding to 1.7 nm in real space, is highlighted (*bold, light blue*), and captures the true displacements well everywhere except at the atomic sites directly adjacent to the boundary. We re-iterate that while a simulated step-function $\pi$ phase slip is useful to evaluate the effectiveness of our approach, energetically we would not expect such high frequency features in experimental PLDs, with the exception of topological defect cores.

*Method Comparison with SrTiO$_3$*

To ensure our Fourier damping approaching was not introducing artificial periodic structure or other artifacts in the lattice displacements, we performed our method on STEM data of cubic $SrTiO_3$ (STO). The results are shown with identical analysis of comparable BSCMO data in Supplementary Figure 14. A single Bragg peak from the Fourier transform of the STO data (Supplementary Figure 14A) shows no satellite peaks, while a single Bragg peak from the Fourier transform of the BSCMO data has two satellite peaks (Supplementary Figure 14D) corresponding to a single PLD modulation in this dataset. After finding the $\mathbf{q}$-vector for the BSCMO data, an equivalent vector scaled to the STO reciprocal lattice was calculated. The peak damping procedure was then performed for both the STO and BSCMO data (Supplementary Figure 14B,E), and PLD maps were generated (Supplementary Figure 14C,F). The scalebar and displacement vector scales are identical for the two PLD maps. The BSCMO data shows periodic stripes of $\sim$10 pm transverse displacements. In the STO data the mean displacement

magnitude is 0.390 pm, 90% of the displacement magnitudes are $\leq 0.83$ pm, 99% of the displacements magnitudes are $\leq 2.08$ pm, and no clear periodicity is observed.

*Atomic fit precision with STO*

In order to determine the precision of our atomic fitting, we fit the positions of all atomic sites in STO data, and calculated distances between neighboring atomic sites. A histogram of the distances between the fit positions of nearest neighbor Sr atoms and the data used are shown in Supplementary Figure 15A,B. A Gaussian fit to the histogram (Supplementary Figure 15A) has a standard deviation of $\sigma = 2.157$ pm and a full width at half maximum of 5.1 pm. Identical analysis of the dimmer, lower signal-to-noise ratio Ti sites yields values of $\sigma = 2.4$ pm and a FWHM of 5.7 pm. While our precision is comparable to that obtained by others, notably it is significantly worse than the 0.6 pm precision obtained in (*11*). We attribute our lower precision to image acquisition which has been optimized for different purposes. Here, we aimed to obtain high precision atomic fits, large fields of view, and optimal Fourier space sampling for satellite peak damping.

*Derivation of Supplementary Equation 7*

Taking the Fourier transform of an image given by Supplementary Equation 1 with modulated lattice sites defined in Supplementary Equation 5, we find

$$\mathcal{F}I^{\mathcal{Q}}(\mathbf{k}) = \mathcal{F}\left(f(\mathbf{r}) * \sum_{\mathbf{R}\in\mathcal{R}^{\mathcal{Q}}} \delta\left(\mathbf{r}-\mathbf{R}\right)\right) \tag{15}$$

$$= \mathcal{F}\left(f(\mathbf{r})\right)\mathcal{F}\left(\sum_{\mathbf{R}\in\mathcal{R}^{\mathcal{Q}}} \delta\left(\mathbf{r}-\mathbf{R}\right)\right) \tag{16}$$

$$= \mathcal{F}f(\mathbf{k}) \sum_{\mathbf{R}\in\mathcal{R}^{\mathcal{Q}}} \left(\mathcal{F}\left(\delta\left(\mathbf{r}-\mathbf{R}\right)\right)\right) \tag{17}$$

$$= \mathcal{F}f(\mathbf{k}) \sum_{\mathbf{R}\in\mathcal{R}^{\mathcal{Q}}} \left(e^{i\mathbf{k}\cdot\mathbf{R}}\right) \tag{18}$$

Using Supplementary Equation (5),

$$\sum_{\mathbf{R}\in\mathcal{R}^{\mathcal{Q}}} \left(e^{i\mathbf{k}\cdot\mathbf{R}}\right) = \sum_{\mathbf{R}\in\mathcal{R}^{\{0\}}} \exp\left[i\mathbf{k}\cdot\left(\mathbf{R}+\sum_{\mathbf{q}\in\mathcal{Q}}\mathbf{A_q}\sin\left(\mathbf{q}\cdot\mathbf{R}+\phi_{\mathbf{q}}\right)\right)\right] \tag{19}$$

$$= \sum_{\mathbf{R}\in\mathcal{R}^{\{0\}}} \exp\left[i\mathbf{k}\cdot\mathbf{R}\right]\exp\left[i\mathbf{k}\cdot\sum_{\mathbf{q}\in\mathcal{Q}}\mathbf{A_q}\sin\left(\mathbf{q}\cdot\mathbf{R}+\phi_{\mathbf{q}}\right)\right] \tag{20}$$

$$= \sum_{\mathbf{R}\in\mathcal{R}^{\{0\}}} \exp\left[i\mathbf{k}\cdot\mathbf{R}\right]\prod_{\mathbf{q}\in\mathcal{Q}}\exp\left[i\mathbf{k}\cdot\left(\mathbf{A_q}\sin\left(\mathbf{q}\cdot\mathbf{R}+\phi_{\mathbf{q}}\right)\right)\right] \tag{21}$$

The Jacobi-Anger expansion may be written as

$$e^{iz\sin\theta} = \sum_{\alpha=-\infty}^{\infty} J_\alpha(z) e^{i\alpha\theta} \tag{22}$$

where $J_\alpha(z)$ is the $\alpha$'th Bessel function of the first kind. Then

$$\sum_{\mathbf{R}\in\mathcal{R}^{\mathcal{Q}}} \left(e^{i\mathbf{k}\cdot\mathbf{R}}\right) = \sum_{\mathbf{R}\in\mathcal{R}^{\{0\}}} \exp\left[i\mathbf{k}\cdot\mathbf{R}\right] \cdot \prod_{\mathbf{q}\in\mathcal{Q}} \sum_{\alpha=-\infty}^{\infty} J_\alpha\left(\mathbf{k}\cdot\mathbf{A}_\mathbf{q}\right) \exp\left[i\alpha\mathbf{q}\cdot\mathbf{R}\right] \exp\left[i\alpha\phi_\mathbf{q}\right] \tag{23}$$

Expanding the product over the $N$ elements in $\mathcal{Q}$,

$$\sum_{\mathbf{R}\in\mathcal{R}^{\mathcal{Q}}} \left(e^{i\mathbf{k}\cdot\mathbf{R}}\right) = \sum_{\mathbf{R}\in\mathcal{R}^{\{0\}}} \exp\left[i\mathbf{k}\cdot\mathbf{R}\right] \sum_{\alpha_{\mathbf{q}_1}=-\infty}^{\infty} \cdots \sum_{\alpha_{\mathbf{q}_N}=-\infty}^{\infty} \prod_{\mathbf{q}\in\mathcal{Q}} \Bigg( J_{\alpha_\mathbf{q}}\left(\mathbf{k}\cdot\mathbf{A}_\mathbf{q}\right) \exp\left[i\alpha_\mathbf{q}\mathbf{q}\cdot\mathbf{R}\right] \exp\left[i\alpha_\mathbf{q}\phi_\mathbf{q}\right] \Bigg) \tag{24}$$

$$= \sum_{\alpha_{\mathbf{q}_1}=-\infty}^{\infty} \cdots \sum_{\alpha_{\mathbf{q}_N}=-\infty}^{\infty} \left( \sum_{\mathbf{R}\in\mathcal{R}^{\{0\}}} \exp\left[i\mathbf{k}\cdot\mathbf{R}\right] \exp\left[\sum_{\mathbf{q}\in\mathcal{Q}} i\alpha_\mathbf{q}\mathbf{q}\cdot\mathbf{R}\right] \cdot \prod_{\mathbf{q}\in\mathcal{Q}} \left( J_{\alpha_\mathbf{q}}\left(\mathbf{k}\cdot\mathbf{A}_\mathbf{q}\right) \exp\left[i\alpha_\mathbf{q}\phi_\mathbf{q}\right] \right) \right) \tag{25}$$

$$= \sum_{\alpha_{\mathbf{q}_1}=-\infty}^{\infty} \cdots \sum_{\alpha_{\mathbf{q}_N}=-\infty}^{\infty} \left( \sum_{\mathbf{R}\in\mathcal{R}^{\{0\}}} \exp\left[i\left(\mathbf{k}+\sum_{\mathbf{q}\in\mathcal{Q}}\alpha_\mathbf{q}\mathbf{q}\right)\cdot\mathbf{R}\right] \cdot \prod_{\mathbf{q}\in\mathcal{Q}} \left( J_{\alpha_\mathbf{q}}\left(\mathbf{k}\cdot\mathbf{A}_\mathbf{q}\right) \exp\left[i\alpha_\mathbf{q}\phi_\mathbf{q}\right] \right) \right) \tag{26}$$

$$= \sum_{\alpha_{\mathbf{q}_1}=-\infty}^{\infty} \cdots \sum_{\alpha_{\mathbf{q}_N}=-\infty}^{\infty} \left( \sum_{\mathbf{b}\in\mathcal{B}^{\{0\}}} \delta\left(\mathbf{k}-\left(\mathbf{b}-\sum_{\mathbf{q}\in\mathcal{Q}}\alpha_\mathbf{q}\mathbf{q}\right)\right) \cdot \prod_{\mathbf{q}\in\mathcal{Q}} \left( J_{\alpha_\mathbf{q}}\left(\mathbf{k}\cdot\mathbf{A}_\mathbf{q}\right) \exp\left[i\alpha_\mathbf{q}\phi_\mathbf{q}\right] \right) \right) \tag{27}$$

where in the last step we've used the fact that $\sum_{\mathbf{R}\in\mathcal{R}^{\{0\}}} \exp\left[i\mathbf{k}\cdot\mathbf{R}\right] = \sum_{\mathbf{b}\in\mathcal{B}^{\{0\}}} \delta\left(\mathbf{k}-\mathbf{b}\right)$, where $\mathcal{B}^{\{0\}}$ is the reciprocal lattice of Bravais lattice $\mathcal{R}^{\{0\}}$.

Defining

$$\mathcal{M}_{\alpha_\mathbf{q},\mathbf{q}}(\mathbf{k}) \equiv J_{\alpha_\mathbf{q}}\left(\mathbf{k}\cdot\mathbf{A}_\mathbf{q}\right) \exp\left[i\alpha_\mathbf{q}\phi_\mathbf{q}\right] \tag{28}$$

yields Supplementary Equation 7.

## Supplementary Note 2: Coarse Grained Phase Field Extraction

We next describe the Fourier space approach to extract the coarse-grained phase field, $\phi(\mathbf{r})$, associated with the $\mathbf{q}_1$ modulation

$$\boldsymbol{\Delta}^{\mathbf{q}_1} \sim \sin(\mathbf{q}_1 \cdot \mathbf{r} + \phi(\mathbf{r})) \tag{29}$$

We interpret the phase field as deviations of the $\mathbf{q}_1$ modulation from perfect periodicity (i.e. where $\phi(\mathbf{r}) = \phi_0 = \text{const}$). We first Fourier filter regions near a $\mathbf{q}_1$ superlattice peak, typically one near the 200 (002) Bragg peak, using a Gaussian filter with a width $\sigma = L^{-1}$ where $L$ is the coarsening length scale in real space. We obtain a real space image where all periodicities in the image, except for the one associated with the $\mathbf{q}_1$ modulation, are removed. Roughly, the filtered image may be described by

$$\tilde{I}(\mathbf{r}) \sim \sin(\mathbf{q}_1 \cdot \mathbf{r} + \phi(\mathbf{r})) \tag{30}$$

In order to extract $\phi(\mathbf{r})$, we use the phase lock-in technique described in (*12*), where we generate two reference signals $\sin(\mathbf{q}_1 \cdot \mathbf{r})$ and $\cos(\mathbf{q}_1 \cdot \mathbf{r})$ with perfect $\mathbf{q}_1$ periodicity and multiply them by $\tilde{I}(\mathbf{r})$ to get $X(\mathbf{r})$ and $Y(\mathbf{r})$ where

$$\begin{cases} X(\mathbf{r}) = \sin(\mathbf{q}_1 \cdot \mathbf{r}) \sin(\mathbf{q}_1 \cdot \mathbf{r} + \phi(\mathbf{r})) \\ Y(\mathbf{r}) = \cos(\mathbf{q}_1 \cdot \mathbf{r}) \sin(\mathbf{q}_1 \cdot \mathbf{r} + \phi(\mathbf{r})) \end{cases} \tag{31}$$

$$\begin{cases} X(\mathbf{r}) = \frac{1}{2} (\cos\phi(\mathbf{r}) - \cos(2\mathbf{q}_1 \cdot \mathbf{r} + \phi(\mathbf{r}))) \\ Y(\mathbf{r}) = \frac{1}{2} (\sin\phi(\mathbf{r}) + \sin(2\mathbf{q}_1 \cdot \mathbf{r} + \phi(\mathbf{r}))) \end{cases} \tag{32}$$

We subsequently low pass filter $X(\mathbf{r})$ and $Y(\mathbf{r})$ to get rid of the second high frequency terms obtaining:

$$\begin{cases} \tilde{X}(\mathbf{r}) \approx \cos\phi(\mathbf{r}) \\ \tilde{Y}(\mathbf{r}) \approx \sin\phi(\mathbf{r}) \end{cases} \tag{33}$$

The coarse-grained phase is thus given by

$$\phi(\mathbf{r}) = \arctan[\tilde{Y}(\mathbf{r})/\tilde{X}(\mathbf{r})] \tag{34}$$

The coarsening length must be chosen judiciously in order to simultaneously optimize the resolution and signal to noise ratio of the resulting coarse grained phase field.

## Supplementary Note 3: Transverse vs. Longitudinal PLDs in Fourier Space

PLDs have several Fourier space features that are distinct from the Fourier space structure of similar phenomena, including charge density waves and superlattices of atomic species. Distinguishing transverse from longitudinal PLDs is readily accomplished in Fourier space by observing the intensity pattern of the satellite peaks with varying $\mathbf{k}$. For simplicity, consider Supplementary Equation 9 describing the experimentally observed peaks. Using Supplementary

Equation 8, the factors damping each satellite peak are given here by

$$c_{\pm,i} = J_1\left(\mathbf{k}\cdot\mathbf{A}_{\mathbf{q}_i}\right)\exp\left(\pm i\phi_{\mathbf{q}_i}\right) \tag{35}$$

$$|c_{\pm,i}| \approx \mathbf{k}\cdot\mathbf{A}_{\mathbf{q}_i} \tag{36}$$

For some modulation wavevector $\mathbf{q}$, consider the satellite peaks about a Bragg peak $\mathbf{b}_{\parallel}$ parallel to the modulation $\mathbf{q} \parallel \mathbf{b}_{\parallel}$. At the satellite positions $\mathbf{k} \approx \mathbf{b}_{\parallel}$ the damping factor is then $|c_{\pm,\parallel}| \approx \mathbf{b}_{\parallel}\cdot\mathbf{A}_{\mathbf{q}}$. Thus $|c_{\pm,\parallel}|$ is maximal for a longitudinal PLD where $\mathbf{q} \parallel \mathbf{A}_{\mathbf{q}}$, while $|c_{\pm,\parallel}| \approx 0$ and the satellite peaks vanish for a transverse PLD where $\mathbf{q} \perp \mathbf{A}_{\mathbf{q}}$. In contrast, consider the satellite peaks about a Bragg peak $\mathbf{b}_{\perp}$, perpendicular to the modulation vector $\mathbf{q} \perp \mathbf{b}_{\parallel}$. Now $|c_{\pm,\perp}|$ is maximal for a transverse PLD where $\mathbf{q} \perp \mathbf{A}_{\mathbf{q}}$, while $|c_{\pm,\perp}| \approx 0$ and the satellite peaks vanish for a longitudinal PLD where $\mathbf{q} \parallel \mathbf{A}_{\mathbf{q}}$. These cases are illustrated schematically in Supplementary Figure 5. Both the STEM Fourier transforms and diffraction patterns (Supplementary Figure 4) of BSCMO clearly indicate transverse PLDs in BSCMO.

# Supplementary References


1. Jia, C-L., Mi, S-B., Urban, K., Vrejoiu, I., Alexe, M., & Hesse, D., Atomic-scale study of electric dipoles near charged and uncharged domain walls in ferroelectric films. *Nat. Mater.* **7**, 57-61 (2008).

2. Nelson, C.T. *et al*. Spontaneous Vortex Nanodomain Arrays at Ferroelectric Heterointerfaces. *Nano Lett.* **11**, 828-834 (2011).

3. Catalan, G. *et al*. Flexoelectric rotation of polarization in ferroelectric thin films. *Nat. Mater.* **10**, 963-967 (2011).

4. Tang, Y. L. *et al*. Atomic-scale mapping of dipole frustration at 90° charged domain walls in ferroelectric $PbTiO_3$ films. *Sci. Rep.* **4:4115**, 1-9 (2014).

5. Yadav, A.K. *et al*. Observation of polar vortices in oxide superlattices *Nature* **530**, 198-201 (2016).

6. Moon, E.J. *et al*. Spatial control of functional properties via octahedral modulations in complex oxide superlattices. *Nat. Comm.* **5:5710**, 1-7 (2014).

7. Kan, D., Aso, R., Sato, R., Haruta, M., Kurata, H., & Shimakawa, Y. Tuning magnetic anisotropy by interfacially engineering the oxygen coordination environment in a transition metal oxide. *Nat. Mater.* **15**, 432-437 (2016).

8. Liao, Z. *et al*. Controlled lateral anisotropy in correlated manganite heterostructures by interface-engineered oxygen octahedral coupling. *Nat. Mater.* **15**, 425-431 (2016).

9. Wilson, J.A., Di Salvo, F.J., & Mahajan, S. Charge-density waves and superlattices in the metallic layered transition metal dichalcogenides. *Adv. Phys.* **24**, 117-201 (1975).

10. Hovden, R. *et al*. Atomic lattice disorder in charge-density-wave phases of exfoliated dichalcogenides (1T-$TaS_2$). *Proc. Natl. Acad. Sci.* **113**, 1142011424 (2016).

11. Yankovich, A.B. *et al*. Picometre-precision analysis of scanning transmission electron microscopy images of platinum nanocatalysts. *Nat. Comm.* **5:4155**, 1-7 (2014).

12. Lawler, M.J. *et al*. Intra-unit-cell electronic nematicity of the high-$T_c$ copper-oxide pseudogap states. *Nature* **466**, 347-351 (2010).

13. Loudon, J.C., Kourkoutis, L.F., Ahn, J.S., Zhang, C.L., Cheong, S.-W., & Muller, D.A., Valence Changes and Structural Distortions in "Charge Ordered" Manganites Quantified by Atomic-Scale Scanning Transmission Electron Microscopy. *Phys. Rev. B* **99**, 237205 (2007).
